# Supplementary material for: Changes in the Rhizosphere Prokaryotic Community Structure of Halodule wrightii Monospecific Stands Associated to Submarine Groundwater Discharges in a Karstic Costal Area
Source: Microorganisms. 2023 Feb 16;11(2):494. doi: 10.3390/microorganisms11020494 (PMC9963909; doi:10.3390/microorganisms11020494)
Supplement: Supplementary file 1 [file microorganisms-11-00494-s001.zip › microorganisms-2210617-supplementary.pdf]

SUPPLEMENTARY MATERIAL

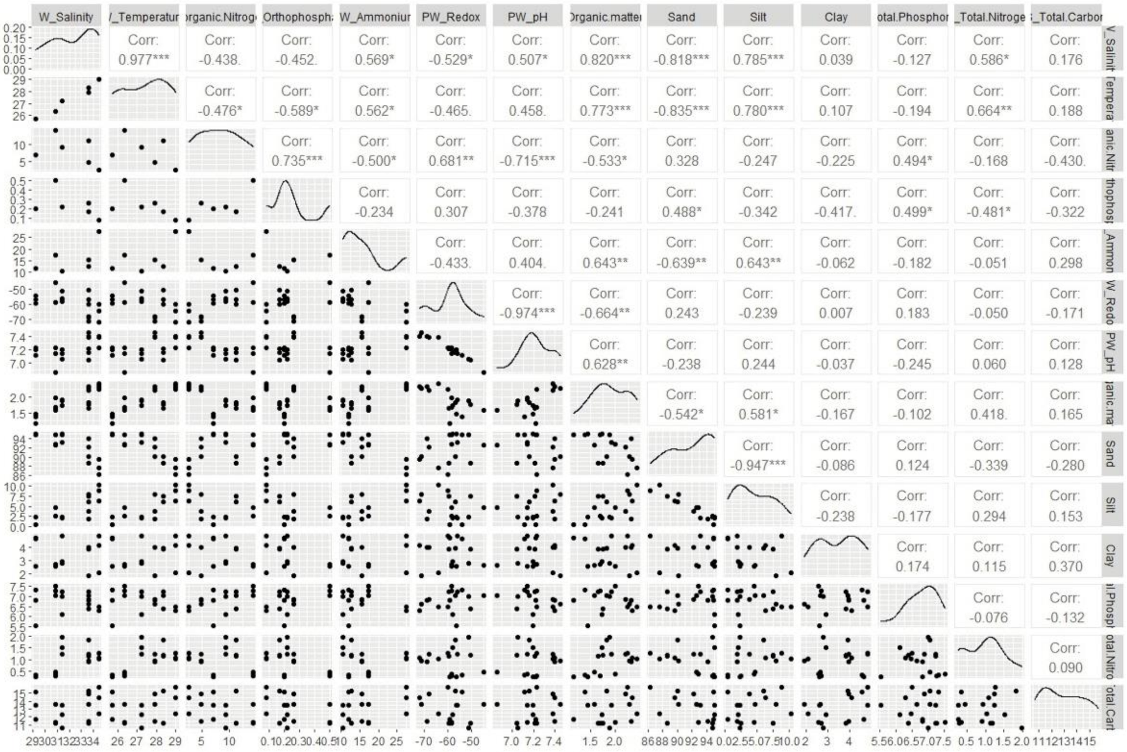

Figure S1. *Autocorrelations, spearman's correlations, and plots between environmental parameters. A single asterisk corresponds to significant data with p-values less than 0.05; a double asterisk corresponds to p-values less than 0.01; and three asterisks correspond to p-values less than 0.001.*

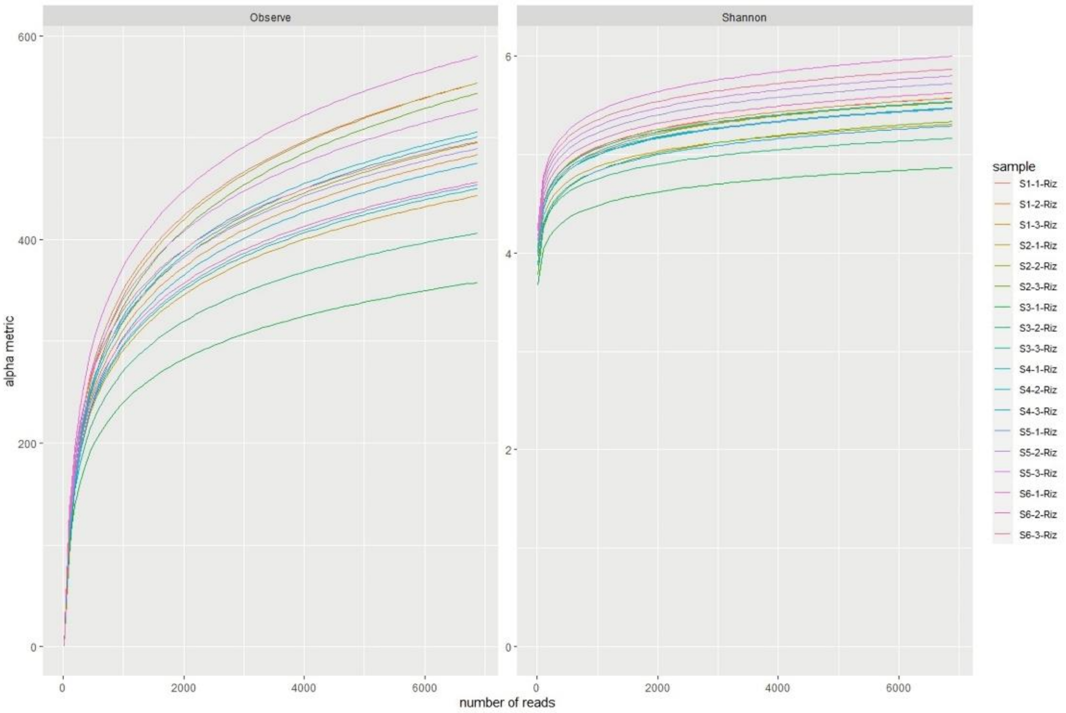

Figure S2. *Rarefaction curves showing Observed and Shannon metrics across sites.*

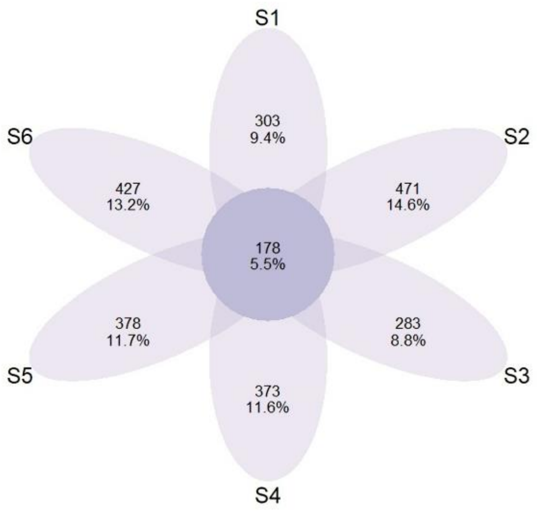

Figure S3. *Six-way petal diagram showing ASV across sites.*

Table S1. *Sampling stations (S1- S6), geographic coordinates and distances from the spring X'Buya-Ha*

Sampling station	Latitude	Longitude	Distance from spring (m)
S1	21°24'18.4" N	88°49'52.8" W	32
S2	21°24'19.7" N	88°49'53.0" W	73
S3	21°24'21.6" N	88°49'52.8O" W	131
S4	21°24'22.9" N	88°49'53.2O" W	171
S5	21°24'25.49" N	88°49'53.32" W	251
S6	21°24'30.46" N	88° 49' 53.70" W	405

Table S2. *Kruskal-Wallis tests followed by Dunn's post tests for environmental characteristics across sites. Only significant factors are presented. A single asterisk corresponds to p-values less than 0.05 and a double asterisk correspond to p-values less than 0.01.*

Type	Parameter	Pairwise comparison	Padj	Significance
Water	Salinity	S2-S6	0.007	**
	Temperature	S2-S6	0.007	**
	Inorganic Nitrogen	S1-S6	0.007	**
	Orthophosphate	S1-S6	0.007	**
Porewater	Ammonium	S3-S6	0.007	**
Sediment	Organic matter	S2-S6	0.015	*
	Sand	S2-S6	0.029	*
	Total nitrogen	S2-S3	0.033	*

Table S3. *Relative abundances for top 10 core community taxa at class and family level. Lowest, Highest and average values with standard deviation are presented.*

		Class	
	MIN	MAX	AVERAGE
Bacteroidia	15.8	28.1	21.7 ± 3.1
Deltaproteobacteria	10.2	21.5	15 ± 2.6
Thermoanaerobaculia	2.9	7	5.2 ± 0.9
Ignavibacteria	3.1	5.2	4.1 ± 0.6
Gammaproteobacteria	4.5	23.3	14.1 ± 6.6
Bathyarchaeia	1.6	6.2	3.4 ± 1.3
Spirochaetia	3.5	5.7	4.5 ± 0.7
Calditrichia	0.7	5.5	3.5 ± 1
Alphaproteobacteria	1.4	5.7	2.3 ± 1
Lokiarchaeia	0.8	3.3	1.6 ± 0.6
		Family	
Flavobacteriaceae	4.7	16.3	8.3 ± 2.4
Desulfobulbaceae	2.3	11.2	5.8 ± 2.3
Bacteroidetes BD2-2	3.7	7.1	5.5 ± 0.9
Thermoanaerobaculaceae	2.9	7	5.2 ± 0.9
Desulfobacteraceae	2.5	8.2	5.9 ± 1.4
Spirochaetaceae	3.5	5.7	4.5 ± 0.7
Calditrichaceae	0.7	5.5	3.5 ± 1
Cyclobacteriaceae	1.9	3.9	3 ± 0.6
PHOS-HE36	1.3	3.1	2 ± 0.5
Sandaracinaceae	0.2	2.2	1.1 ± 0.5

Table S4. *Relative abundances for top 10 core community taxa at genus level. Lowest, highest, and average values with standard deviation are presented.*

	Genus		
Subgroup 23	2.4	5.9	4.5 ± 0.8
Actibacter	2.6	8.9	4 ± 1.4
Robiginitalea	1.4	4.4	2.9 ± 0.7
Spirochaeta 2	2.4	4	3.1 ± 0.5
Sva0081 sediment group	0.7	2.4	1.8 ± 0.5
Desulfococcus	0.3	1.2	0.9 ± 0.2
Woeseia	0.7	2.5	1.5 ± 0.5
Ruegeria	0.3	1.5	0.6 ± 0.3
Candidatus thiodiazotropha	0.1	1.1	0.7 ± 0.2
Ignavibacterium	0.2	0.6	0.4 ± 0.1
